# Anomalous Mode Transitions in High Power Distributed Bragg Reflector Quantum Cascade Lasers

**DOI:** 10.1186/s11671-019-3151-3

**Published:** 2019-10-22

**Authors:** Feng-Min Cheng, Jin-Chuan Zhang, Zeng-hui Gu, Dong-Bo Wang, Ning Zhuo, Shen-Qiang Zhai, Li-Jun Wang, Jun-Qi Liu, Shu-Man Liu, Feng-Qi Liu, Zhan-Guo Wang

**Affiliations:** 1 Key Laboratory of Semiconductor Materials Science, Institute of Semiconductors, Chinese Academy of Sciences, Beijing Key Laboratory of Low Dimensional Semiconductor Materials and Devices, P.O. Box 912, Beijing, 100083 China; 2 Center of Materials Science and Optoelectronics Engineering, University of Chinese Academy of Sciences, Beijing, 100049 China; 3 Beijing Academy of Quantum Information Sciences, Beijing, 100193 China

**Keywords:** Anomalous mode transitions, Quantum cascade laser, Distributed Bragg reflector

## Abstract

In this paper, an anomalous spectral data of distributed Bragg reflector (DBR) quantum cascade lasers (QCLs) emitting around 7.6 μm is presented. The two-section DBR lasers, consisting of a gain section and an unpumped Bragg reflector, display an output power above 0.6 W in continuous wave (CW) mode at room temperature. The anomalous spectral data is defined as a longitudinal mode which moves toward shorter wavelengths with increasing temperature or injection current, which is unexpected. Although the longer wavelength modes are expected to start lasing when raising device temperature or injection current, occasional mode hops to a shorter wavelength are seen. These anomalous mode transitions are explained by means of modal analysis. The thermal-induced change of the refractive index implied by an increase in the temperature or injection current yields nearly periodic transitions between cavity modes.

## Introduction

Quantum cascade lasers (QCLs) differ from fundamental semiconductor lasers, are a kind of unipole semiconductor laser, namely, electronic transitions only between the conduction band states [1]. It has attracted much attention owing to its highlight features of large wavelength covering the range from the mid-/far-infrared to the terahertz wave region ever since its first demonstration in experiment. Such wide wave region can meet the increasing demands of applications in gas sensing, high-resolution spectroscopy, and industrial process monitoring. However, a narrow linewidth and high output power are required in some applications. Distributed feedback (DFB) QCLs and external cavity (EC) QCLs are the two common ways for achieving single-mode emission [2, 3]. The output power of DFB QCLs is in the order of 100 milliwatts and the tuning range is small about 5 cm^−1^, which is suitable for single gas detection [4–6]. EC QCLs are better candidates for detection of multiple gas species because they have much broader tuning range [7]. However, in some applications, such as stand-off detection or remote sensing, a high power single-mode light source is desired. For these applications, a distributed Bragg reflector (DBR) QCL may be a better candidate as compact and high power laser source. DBR lasers were studied quite much on near-infrared wave region [8–10], but its study on QCL is less, few were reported in 2011 for wide tuning [11], in 2014 for high power [12]. However, the spectral properties were not studied in detail in those reports. Furthermore, this kind similar anomalous mode hops have been analyzed in near-infrared (IR) DBR semiconductor lasers [9, 10]. However, it is still lacking in QCL device. Considering the spectral properties of single-mode QCLs are significant for practical applications, any anomalous and unexplored properties should be extensively studied and accumulated. Here, we demonstrate DBR QCLs and investigate their spectral properties in detail.

## Methods

**Fig. 1:**
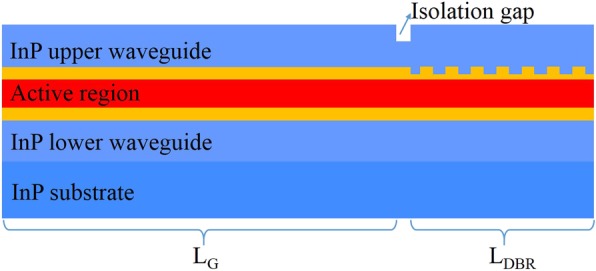
Scheme of a DBR QCL consisting of a gain section *L*_G_, a DBR section *L*_DBR_ and a current isolation gap

The DBR grating was defined by conventional double beam holographic interferometry process. The designed device structure is shown in Fig. 1. The gain section and the DBR section were separated by a current isolation groove and only the gain section has a current injection. The QCL structure was grown on an n-doped InP substrate by solid-source molecular beam epitaxy (MBE) with an active-region structure similar to Ref. [13]. The active core structure presented in this work contains 50 periods of strain-compensated In_0.58_Ga_0.42_As/In_0.47_Al_0.53_As quantum wells. The specific layer sequence of one period is as follows (layer thickness in nanometers): **4**/1.7/**0.9**/5.06/**0.9**/4.7/**1**/3.9/**1.8**/3.2/**1.7**/2.8/**1.9**/*2.7*/***2.8***/2.6, where In_0.47_Al_0.53_As barrier layers are in bold, In_0.58_Ga_0.42_As well layers are in roman, and n-doped layers (1.4 × 10^17^ cm^−3^) are in italic. The whole wafer structure before the fabrication is 4.5 μm InP lower cladding layer (Si, 3 × 10^16^ cm^−3^), 50 active/injector stages, 0.3-μm-thick n-In_0.53_Ga_0.47_As upper confinement layer (Si, 4 × 10^16^ cm^−3^). A 100-nm-thick SiO_2_ layer was deposited in the upper InGaAs confinement layer in whole wafer, then the SiO_2_ of DBR section was removed for fabricating grating. After that, the grating was defined on the upper InGaAs confinement layer using double beam holographic interferometry process with a grating period of 1.2 μm, then transferred by wet chemical etching to the depth of about 130 nm, subsequently the residual SiO_2_ was removed. Then, the top waveguide consisting of a 3 μm thick upper InP cladding layer (Si, 2 × 10^16^ cm^−3^), 0.15 μm gradually doped InP layer (Si, 1.5 × 10^17^ cm^−3^) and a 0.85 μm thick upper highly doped InP contact layer (Si, 5 × 10^18^ cm^−3^) was regrown by metal-organic vapor phase epitaxy (MOVPE).

After the implementation of regrowth, the wafer was processed into double-channel ridge waveguide laser with an average core width of 10 μm, where the channels were filled with semi-insulating InP:Fe for the purposes of effective thermal dissipation and electrical insulation. Next, a 200-μm-long current isolation groove between the gain section and the DBR section was etched through the upper highly doped and gradually doped InP layer with a depth of 1.1 μm via dry etching for blocking current injecting into the DBR section. Then an insulation layer of 450 nm-thick SiO_2_ was deposited, and the current injection window was opened just on top of the gain section. Subsequently, electrical contact was provided by a Ti/Au layer deposited by electron beam evaporation, and an additional 5-μm-thick gold layer was electroplated for further improving heat dissipation. After being thinned down to about 120 μm, a Ge/Au/Ni/Au metal contact layer was deposited on the substrate side of the wafer. Finally, the wafer was cleaved into 6-mm-long devices consisting of 4.3-mm-long gain region, 1.5-mm-long DBR region and 0.2-mm-long current isolation groove, and the devices were soldered epilayer side down onto the diamond heat-sink with indium solder, which were subsequently soldered on copper heat sinks.

## Results and Discussion

The spectra of devices were tested by a Fourier transform infrared spectrometer with a resolution of 0.125 cm^−1^. The power–current–voltage (P–I–V) characteristics of the devices were tested by a calibrated thermopile detector. The laser was mounted on a holder containing a thermistor combined with a thermoelectric cooler to monitor and adjust the sub-mount temperature. The emitted optical power was measured with the calibrated thermopile detector placed in front of the laser facet without any correction.

**Fig. 2:**
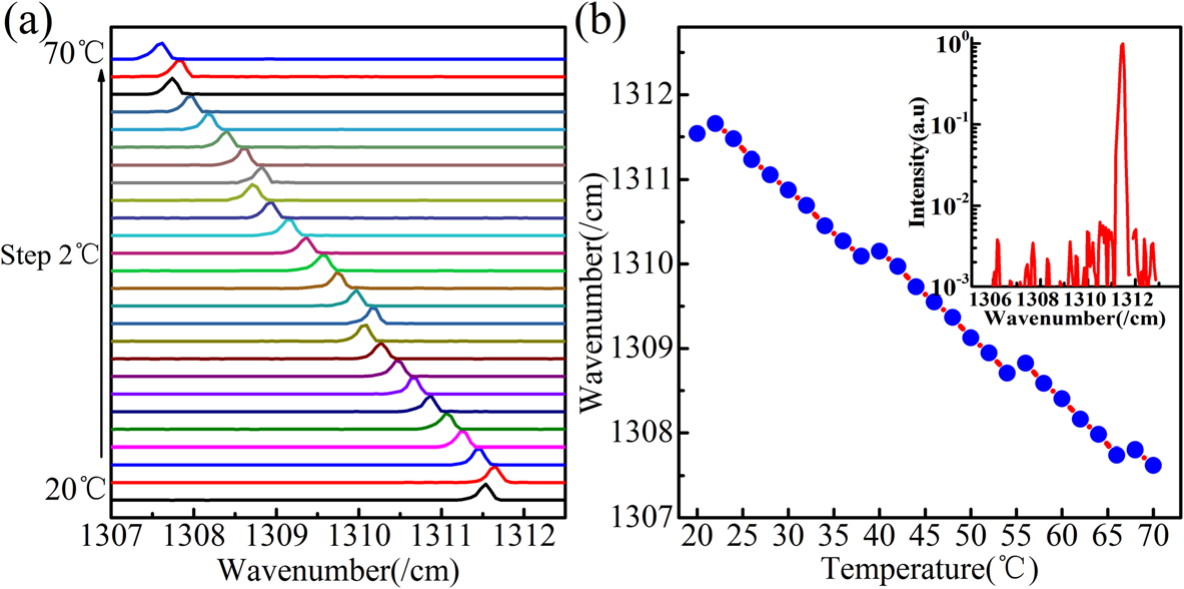
**a** The emission spectra of laser changing with temperature. **b** The wavenumber versus temperature curve of the laser. The inset shows lasing spectrum of 24 °C by logarithmic coordinate

Figure 2a shows the continuous wave (CW) emission spectra of DBR laser at different heat sink temperatures from 20 °C to 70 °C with a step of 2 °C with injection currents of 1.005I_th_. Figure 2b shows the wavenumber versus temperature curve of laser, and the inset shows a lasing spectrum of 24 °C by logarithmic coordinate, where the side-mode suppression ratio (SMSR) is about 25 dB. In conventional single-mode DFB QCLs, the wavelengths shift toward longer wavelengths linear with the increase of temperature or current [14, 15]. However, as ones have seen from Fig. 2, an anomalous tuning behavior is observed, with mode hops toward shorter wavelengths when the temperature raising.

**Fig. 3:**
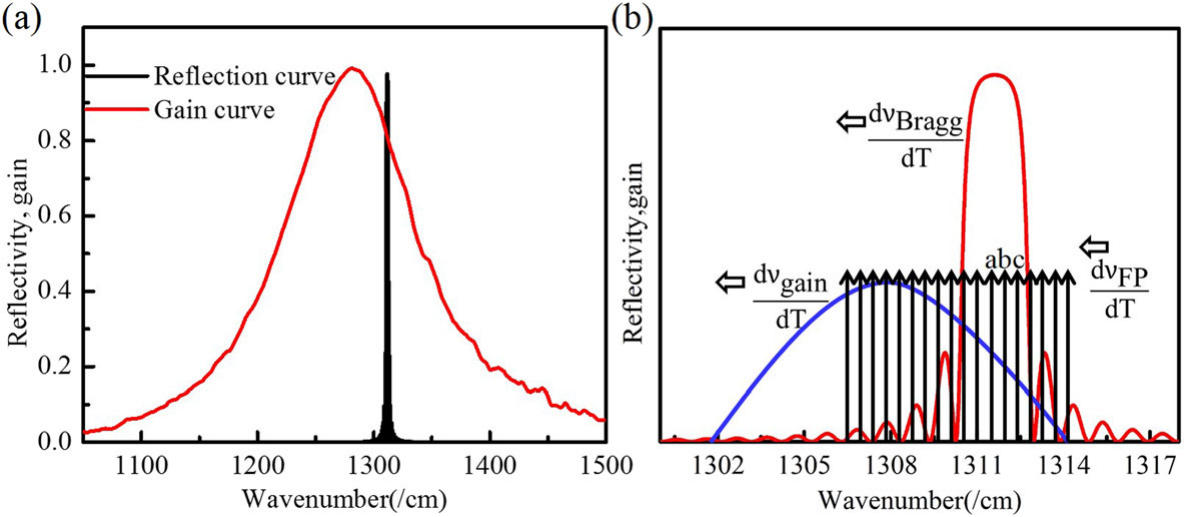
**a** The measured wafer gain curve and calculated the reflection curve of Bragg grating based on transfer matrix method by MATLAB. **b** The schematic diagram of mode selection mechanism of DBR QCL

**Fig. 4:**
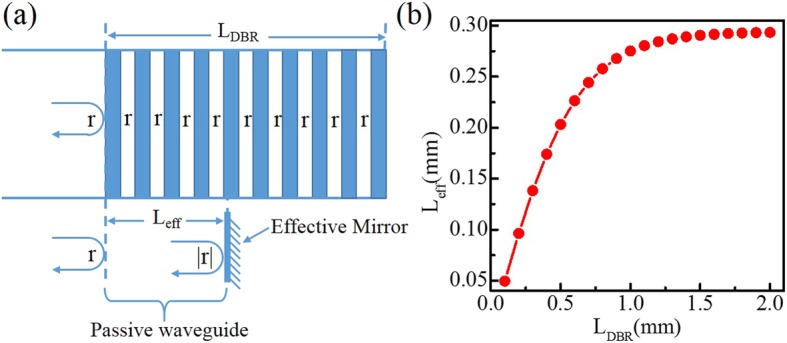
**a** The schematic diagram of definition of an effective mirror for a DBR grating. **b** The effective DBR length versus the physical grating length

To qualitatively explain the occurrence of the anomalous mode hops, first, we need to analyze the mechanism of single-mode at the DBR QCL device, which is shown clearly in Fig. 3. Figure 3a indicates the measured wafer gain curve and calculated the reflection curve of Bragg grating based on transfer matrix method by MATLAB, where the reflectivity of 1.5-mm-long DBR grating is about 98%. For understanding easily, we display the schematic diagram of the mode selection mechanism of DBR QCL, where the gain curve, the reflection curve of Bragg grating, allowed longitudinal modes which satisfy the phase condition in the DBR QCL cavity are displayed, and give their shift characteristics with the increase of temperature in Fig. 3b further. Which one longitudinal mode can be the lasing mode among these longitudinal modes? It should meet two conditions, first it should locate within the Bragg reflection peak. Another condition is that its product of value of gain and reflectivity should be maximum [9]. Furthermore, the gain curve, the reflection curve and longitudinal modes spectra are all moving to longer wavelength with the increase of temperature. Then we measured and fitted the curve of gain peak with the change of temperature to attain the moving rate of − 0.581 cm^−1^ K^−1^. The Bragg reflection peak with the increase of temperature is about − 0.128 cm^−1^ K^−1^ according to our group previously reported result at similar wave range [16]. That is to say, the Bragg reflection peak always remains on the shorter wavelength side of the gain peak as the temperature is increasing. The longitudinal modes spectra movement as the increase of temperature is mainly attributed to the modal refractive index growing with the increase of temperature, whose moving rate is similar to the moving rate of Bragg reflection peak with the increase of temperature smaller than the moving rate of gain peak. However, the temperature of gain region is slightly higher than that of DBR region due to the heat effect caused by carrier injection. As a result, the longitudinal modes spectra can move slightly faster than the Bragg peak with the increase of temperature. We number the three longitudinal modes as a, b, and c within the Bragg peak in Fig. 3b. At start, the mode a was the lasing mode, and the mode a would tune linearly and shift toward longer wavelength with the increase of temperature. The lasing mode would be replaced by mode b when the mode a shifted away from the center of Bragg curve and its product of value of gain and reflectivity was no longer maximum due to the slightly faster moving rate of longitudinal modes spectra. Then, the mode b repeated the process of mode a, and so on. So the phenomenon of anomalous mode hops in Fig. 2 is observed. For verifying the mode hop is between longitudinal modes. Then we calculated the longitudinal mode spacing, which is relative to the whole effective cavity length of device. The whole effective cavity length of DBR QCL is the sum of the effective DBR section length, the gain section length, and the isolation gap length. The definition of effective DBR length is that note the phase varies relatively linearly near the reflection maximum. Such a reflection can be well approximated by a discrete mirror reflection equal to the magnitude of the grating’s reflection, but placed a distance *L*_eff_ away as shown in Fig. 4a. That is to say, the function of whole DBR grating is replaced by a reflection mirror, which is equivalent to a passive waveguide. The effective DBR length of physical DBR grating length can be calculated based on the following equations [17]:

1 $$ {L}_{\mathrm{eff}}=\frac{1}{2\upkappa}\tanh \left({\upkappa \mathrm{L}}_{\mathrm{DBR}}\right) $$

2 $$ \upkappa =\frac{1}{\Lambda}\frac{\Delta \overline{\mathrm{n}}}{\overline{\mathrm{n}}} $$

where κ is the grating coupling coefficient and *L*_DBR_ is the physical grating length, $\Delta \overline{\mathrm{n}}$ is the effective refractive index difference of grating, $\overline{\mathrm{n}}$ is the average effective refractive index of grating, and Λ is the period of grating. Figure 4b shows the effective length of DBR region versus physical DBR grating length, which indicates the effective DBR length almost no longer changed when the physical DRB grating length was larger than 1.5 mm. The effective DBR length of 1.5-mm physical DBR grating length is about 0.291 mm. As a result, the theoretical longitudinal mode spacing is about 0.328 cm^−1^. The experimental anomalous mode hops interval is about 0.12 cm^−1^ shown in Fig. 2. The average linear tuning characteristic of lasing mode with temperature is about 0.103 cm^−1^ K^−1^ before anomalous every mode hops happen. So the corresponding mode spacing is 0.326 cm^−1^, which is almost consistent with the calculated result of 0.328 cm^−1^ with the error of − 0.002 cm^−1^.

**Fig. 5:**
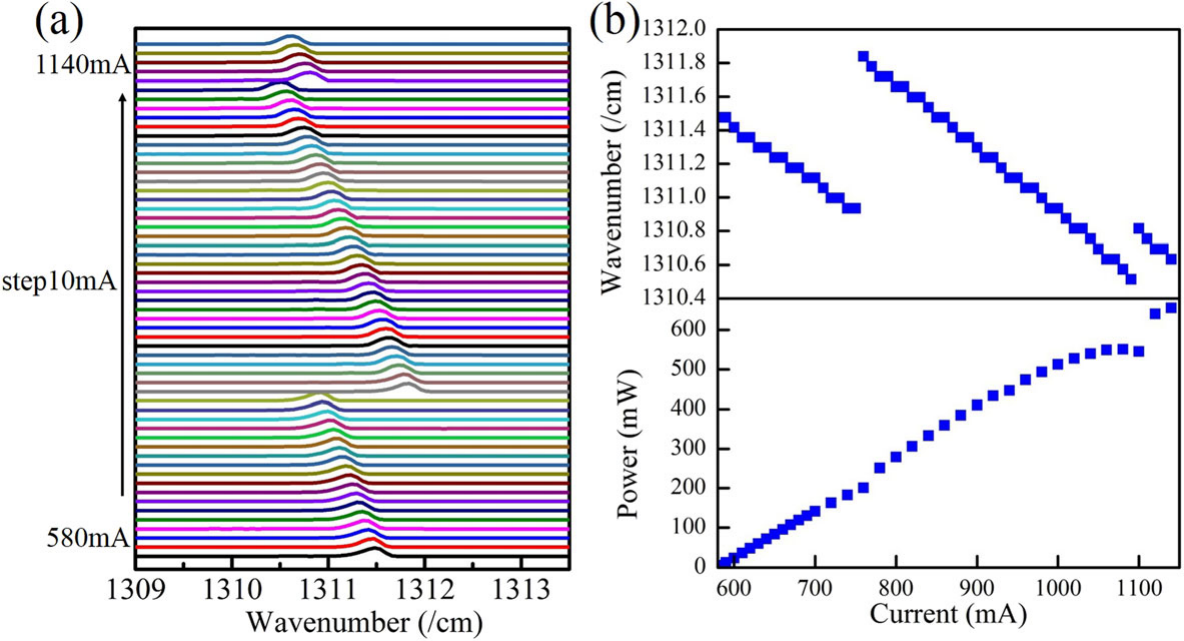
**a** The emission spectrum changing with the injection current. **b** The top panel shows the wavenumber versus temperature curve, and the bottom panel is the CW power–current (P–I) curve of laser

Figure 5a shows the emission spectra changing with injection current, the top panel of Fig. 5b shows the wavenumber versus temperature curve of the device, and the bottom panel of Fig. 5b is the CW P–I curve of laser. The similar phenomenon of anomalous mode hops is also observed with the increase of injection current in Fig. 5, and the obvious discontinuity is seen in P–I curve when the mode hops happen, which cannot happen in conventional single-mode DFB QCLs except for occasional mode hop between the two side-mode of stop-band. The gain peak would always shift toward longer wavelength with the increase of injection current due to heat effect. We have measured the gain curve of wafer changing with the current at CW condition, and fitted curve of gain peak with the change of current to obtain the moving rate of − 0.021 cm^−1^ mA^−1^. Because the current injection window was opened just on top of the gain region and the existence of isolation gap, the influence of heat crosstalk caused by current injection on the DBR section is weak. So the Bragg reflection curve almost does not change with the injection current. The longitudinal modes spectrum also moves slightly to longer wavelength due to the change of modal effective refractive index caused by heat effect. Hence, the similar phenomenon of anomalous mode hops is observed when injection current raising. The first anomalous mode hop interval is about 0.904 cm^−1^ shown in Fig. 5, where the mode hop crossed to three longitudinal modes. The second mode hop is between neighboring longitudinal modes with the interval of 0.301 cm^−1^. The average linear tuning characteristic of lasing mode with injection current is about − 0.003 cm^−1^ mA^−1^ before every anomalous mode hops happen. So the corresponding mode spacing is around 0.331 cm^−1^, which is also almost consistent with the calculated result of 0.328 cm^−1^ with the error of 0.003 cm^−1^.

**Fig. 6:**
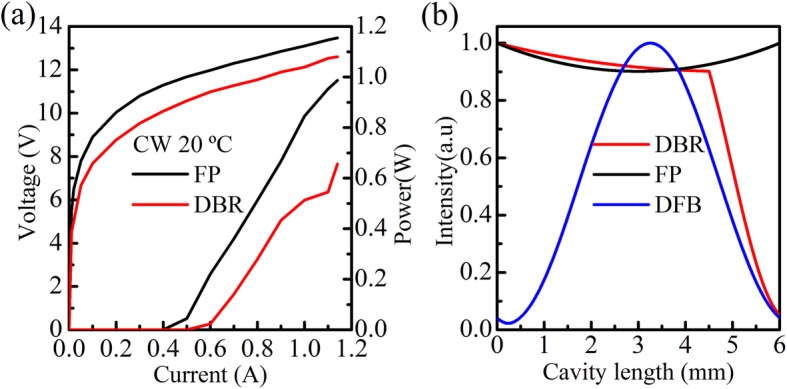
**a** The red curve is the CW power–current–voltage (P–I–V) curve of DBR laser, the black curve is CW power–current–voltage (P–I–V) curve of the Fabry-Perot (FP) resonator cavity. **b** The simulated optical field distribution of DBR, FP and DFB lasers with the same cavity length of 6 mm

Figure 6a shows the comparison of power between DBR laser and Fabry-Perot (FP) laser with a cavity length of 4 mm. The maximum output power of FP and DBR laser is 987 mW and 656 mW at 20 °C, respectively. The maximum output power of DBR laser is still 235 mW at the heat sink temperature of 70 °C, which represented the highest power level reported so far for any single-mode QCLs in the longwave infrared spectrum range. To avoid damage, lasers were not tested to their maximum working current. Figure 6b displays the optical field distribution of DBR, FP, and DFB lasers with the same cavity length of 6 mm based on transfer matrix method by MATLAB. The optical field distribution of DBR laser indicates that the intensity of the light in the device is nearly constant in the gain section similar to the FP laser while it decreases sharply in the DBR section, which is in favor of the power extracting not like DFB laser, whose light intensity peaks in the center of the device and decays rapidly toward the two ends facets due to the over-coupled mechanism, as a result, most light intensity is limited in the center of device. The coupled strength of DFB laser is directly proportional to cavity length. So DFB laser is not suitable to be cleaved into long cavity length device. As a result, the other prominent advantage of DBR laser is presented that it can be fabricated in long cavity length device for obtaining high output power. So the DBR structure is a kind of potential method to achieve high power and single-mode emission.

## Conclusions

In summary, we have demonstrated DBR QCLs with high output power. The mode hops characteristics have been analyzed in detail, where this research is useful for practical applications. The maximum CW output power is 656 mW at 20 °C for the device with 4.3-mm-long gain region. From the comparison of optical field distribution and our analyzed results, we conclude that DBR is a potential and effective method for QCLs to achieve high output power and single-mode emission.

## Data Availability

All data are fully available without restriction.

## Abbreviations

CW: Continuous wave
DBR: Distributed Bragg reflector
DFB: Distributed feedback
EC: External cavity
FP: Fabry-Perot
MBE: Molecular beam epitaxy
MOVPE: Metal organic vapor phase epitaxy
P–I–V: Power–current–voltage
QCL: Quantum cascade laser
SMSR: Side mode suppression ratio
